# Comparing the Visual Analogue Scale and the Pediatric Quality of Life Inventory for Measuring Health-Related Quality of Life in Children with Oral Clefts

**DOI:** 10.3390/ijerph110404280

**Published:** 2014-04-16

**Authors:** George L. Wehby, Hodad Naderi, James M. Robbins, Timothy N. Ansley, Peter C. Damiano

**Affiliations:** 1 Departments of Health Management and Policy, Economics, and Preventive and Community Dentistry, and Public Policy Center, University of Iowa, 145 N. Riverside Dr. 100 College of Public Health Building, Room N248, Iowa City, IA 52242, USA; 2 Roy J. and Lucille A. Carver College of Medicine, University of Iowa, Iowa City, IA 52242, USA; E-Mail: hodad-naderi@uiowa.edu; 3 Departments of Pediatrics and Psychiatry, University of Arkansas, 1 Children’s Way, Little Rock, AR 72202, USA; E-Mail: RobbinsJamesM@uams.edu; 4 Psychological and Quantitative Foundations, University of Iowa, 314 LC, Iowa City, IA 52242, USA; E-Mail: timothy-ansley@uiowa.edu; 5 Department of Preventive and Community Dentistry and Public Policy Center, University of Iowa, 212 SQ, Iowa City, IA 52242, USA; E-Mail: peter-damiano@uiowa.edu

**Keywords:** oral clefts, quality of life, visual analogue scale, child development, birth defects

## Abstract

*Objectives*: To evaluate the performance of the Visual Analogue Scale (VAS), in measuring overall health-related Quality of Life (HRQoL) in children with oral clefts relative to the Pediatric Quality of Life Inventory 4.0 (PedsQL^TM^) Generic Core Scales, one of the most validated and commonly used methods to measure pediatric HRQoL. *Methods*: The study included a population-based sample of 307 children aged 5 to 10 years who were born in Iowa, New York, and Arkansas with non-syndromic oral clefts. Data on HRQoL were obtained using a VAS and PedsQL^TM^ via self-administered interviews with the parents. We evaluated the correlations between the VAS and PedsQL^TM^ total scores, and the correlations of each of these two scales with a series of child health and wellbeing indicators. *Results*: The VAS and PedsQL^TM^ scores were well-correlated (*r* = 0.67). There were no prominent differences between the correlations of VAS and PedsQL^TM^ with the selected indicators of child health and wellbeing; differences in correlations were less than 0.1. Differences in HRQoL by cleft type were more pronounced on the PedsQL^TM^. *Conclusions*: Our study finds the VAS to perform relatively well in measuring overall HRQoL among children with oral clefts. The VAS may be useful as a screening tool to identify children with oral clefts at risk of low HRQoL for referral into more comprehensive evaluations and for measuring average HRQoL across a sample of children.

## 1. Introduction

Oral clefts are one of the most common birth defects worldwide, and have lifelong implications for the wellbeing and health-related quality of life (HRQoL) of affected children and their families [1,2,3]. Among the many potential consequences early in life and during childhood are increased risks for fetal growth retardation [4], hospitalizations [5] and certain behavioral and psychosocial problems such as inattention/hyperactivity and separation anxiety disorder [6,7], which may be partly due to concerns about facial appearance and speech but also other factors such as the multiple needed surgical repairs and healthcare treatments [6]. 

Health-related quality of life (HRQoL) has become a commonly used measure of health and well-being that represents the impact of health on quality of life and captures the desirability of health conditions relative to perfect health. In addition to being a powerful measure of health status, preference-based HRQoL measures can be used to adjust duration of life to generate quality-adjusted life years (QALYs), a standard measure of effectiveness in cost-utility analyses used for assessing the value of health care treatments and resources to society relative to cost [8]. Therefore, measuring the HRQoL of children with oral clefts is of interest for both researchers and clinicians given the greater risk of affected children for a decline in HRQoL compared to the general population due to both physical and psychosocial effects and the increased need for interactions with healthcare professionals including pediatricians. 

Several methods have been suggested for measuring HRQoL including instruments that have been developed to measure HRQoL in children. Of these, the 23-item Pediatric Quality of Life Inventory 4.0 (PedsQL^TM^) Generic Core Scales is one of the most commonly utilized. The PedsQL^TM^ is a short survey (estimated to take less than 5 min) designed to measure HRQoL for children aged 2 to 12 and adolescents 13-18 years through questions related to the physical, emotional, and social functioning and has high feasibility, reliability and validity [9,10,11,12]. 

Visual Analogue Scale (VAS) methods that involve direct rating and state comparisons can also be used to measure HRQoL [1,13]. Unlike instruments that involve answering a series of direct questions about health and well-being, this method asks an individual to rate health status or a particular health state on a scale between two reference states/points (typically death and perfect health) that represents the desirability of that health status or state relative to these states. The simplicity and ease of administering VAS methods allow for their wide use in many different settings [1,14]. The reliability, validity, and feasibility of direct rating methods have been demonstrated in the literature [14,15,16]. 

Given the availability of different methodologies to measure HRQoL and the importance of measuring HRQoL among children with oral clefts, the question arises as to how different methodologies such as the VAS and the PedsQL^TM^ compare to each other in this population. The benefits of VAS include quick adaptability in both research and clinical settings and that VAS-based HRQoL scores can be easily used to obtain quality-adjusted- life-years (QALYs) for cost-effectiveness analysis. There are also limitations with the VAS, including potential bias in measurement due to raters avoiding the ends of the scale or with measuring multiple co-existing health conditions [17]. Another limitation is that the VAS is generally designed to obtain a single HRQoL score for overall health status without additional detail on the various domains of health. While this disadvantage may be arguably overcome by constructing VAS for each of the main domains of health including, physical, social, and mental health, this may take away from the practical advantage of the VAS. In general, VAS may be particularly appealing in settings where the primary goal is to screen for low HRQoL or to obtain overall HRQoL across a sample relatively easily and at little cost. Given that the PedsQL^TM^ is one of the most validated and commonly used instruments for pediatric HRQoL, this study compares the performance of the VAS relative to the PedsQL^TM^ in measuring the HRQoL of children with oral clefts and the consistency of the HRQoL scores between the two methods.

## 2. Methods

### 2.1. Data

The data from this study were obtained from a mail survey conducted with the parents of 307 children age 5 to 10 years who were born with non-syndromic oral clefts in Iowa, New York, and Arkansas. Data were obtained on HRQoL using both the PedsQL^TM^ and VAS measures of HRQoL. The survey was conducted in 2007–2008; the children were required to be currently living in one of the three aforementioned states with a parent. A written survey using a modified Dillman method was mailed asking questions about a wide range of topics [18]. First a survey questionnaire was sent along with a letter discussing the purpose of the study. A week later, a postcard was sent as a means to remind participants to return in their surveys if they wished to participate. If a participant did not respond within 10 days, then another survey questionnaire and letter were sent. All necessary Institutional Review Boards approved the study.

### 2.2. Comparison of HRQL Instruments

The mothers were asked to complete the parental version of the PedsQL^TM^ for their children. In addition, they were asked to rate their child’s HRQoL on a VAS:

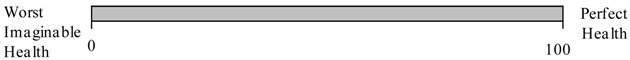



Specifically, each mother was asked to draw a vertical line (|) at the point on the scale that she thought represented the status of her child’s HRQoL. The VAS score was calculated as the distance between the left anchor of the scale (0 value or worst imaginable health) and the vertical line drawn by the mother. The scale was described as follows:

*On the scale below, we ask you to rate your child’s health-related quality of life on a scale of 0 to 100. A score of “0” represents the worst health state that you can imagine. A score of “100” represents perfect health. A child with perfect health would be one who has no pain or discomfort, no anxiety or depression, and no problems with usual activities that would be expected for his or her age, such as feeding him or herself, speaking, playing with other children, washing his or her hands, participating in school activities.*

We first evaluated the correlations between the VAS and PedsQL^TM^ total scores. Next, we evaluated the correlations of each of these two scales with a series of child health and wellbeing indicators in order to evaluate if any the two was more strongly correlated with these measures. For each of these indicators, we calculated the correlations for the subgroup that had complete (non-missing data) on the indicator and on both the VAS and the PedsQL^TM^ (11 observations had missing data on one or both of these scales). Measures of the child’s social and separation anxiety were obtained using the 41-item Screen for Child Anxiety Related Emotional Disorders (SCARED) [19]. A subscale of 8 SCARED items make up the separation anxiety score which has a maximum of 16, with a score of 5 or greater indicating separation anxiety disorder. Similarly, the social anxiety variable consists of a subscale of 7 items from the SCARED items with a maximum score of 14, with a score of 8 or greater indicating social anxiety disorder. Data were also obtained on the Pediatric Behavior Scale (PBS), a 30 item survey which focuses on four broad areas of depression/anxiety, physical/somatic symptoms, aggression/opposition, and inattention/hyperactivity [6]. Other indicators of the child’s health and well-being included maternal rating of the child’s overall health status on a standard Likert-scale, whether the child suffered from a chronic health condition (under 25 categories such as asthma, vision, dental, hearing, and other problems), and how happy the child was with his or her facial appearance on a four-category scale, a commonly used and particularly relevant measure for this population [6]. Additionally, five variables focused on aspects of how the child’s condition affected his or her ability to be understood while speaking. Table 1 includes the definitions of the study variables.

## 3. Results

Out of 589 eligible children, questionnaires were received from 307, yielding a response rate of 52.1%. About 62% of the sample were males (which is expected since oral clefts are more common among males) and 92% were Caucasian. The sample was approximately evenly distributed across the ages of four to nine with a range of 42 to 58 children in each year. The rates of cleft type were overall comparable to population rates in the US including 81 children (28%) with cleft lip only (CLO), 95 children (30%) with cleft palate only (CPO), and 131 children (42%) with both cleft lip with palate (CLP). These statistics suggest no response bias over child’s gender, race/ethnicity, age, and cleft type.

**Table 1:** Variable Description and Descriptive Statistics.

Health Measure	Variable Name (for Reference)	N	Mean	Standard Deviation	Minimum	Maximum
visual analog scale	VAS	301	86.6	15.9	20	100
Pediatric Quality of Life Inventory 4.0	PedsQL	301	83.8	16.4	25	100
In general, how would you rate your child’s overall health now? (1 = excellent, 5 = poor)	child's overall health	300	1.55	0.80	1	5
If child has any chronic health condition under one or more of 25 categories (e.g., asthma, attention, vision, hearing, heart, muscle, or other problems) then the value is = 1 otherwise, it is = 0	chronic condition	307	0.82	0.38	0	1
How often does your child appear to get frustrated when he or she speaks because of trouble being understood? (1 = never, 4 = always)	child frustrated because of trouble being understood	301	1.58	0.70	1	4
How often does your child appear to avoid talking because of trouble being understood? (1 = never, 4 = always)	child avoids speaking	302	1.21	0.51	1	4
How often does your child appear to have difficulty being understood by the people who are with him or her every day? (1 = never, 4 = always)	difficulty being understood by those who do see child often	303	1.48	0.64	1	4
How often does your child appear to have difficulty being understood by people who don’t interact with or see him or her very much? (1 = never, 4 = always)	difficulty being understood by those who do not see child much	302	1.82	0.86	1	4
Overall, how happy would you say your child is with his or her facial appearance? (1 = very happy, 4 = not happy at all)	happy with facial appearance	298	1.34	0.62	1	4
Social anxiety (Question 15)—binary variable	social anxiety	307	0.16	0.36	0	1
Separation anxiety (Question 15)—binary variable	separation anxiety	307	0.25	0.44	0	1
PBS aggression/opposition	PBS aggression/opposition	296	4.66	4.39	0	27
PBS hyperactivity/inattention	PBS hyperactivity/inattention	297	7.27	6.95	0	27
PBS depression/anxiety	PBS depression/anxiety	297	2.08	3.09	0	16
PBS physical health	PBS physical health	299	1.12	1.92	0	13

**Figure 1:**
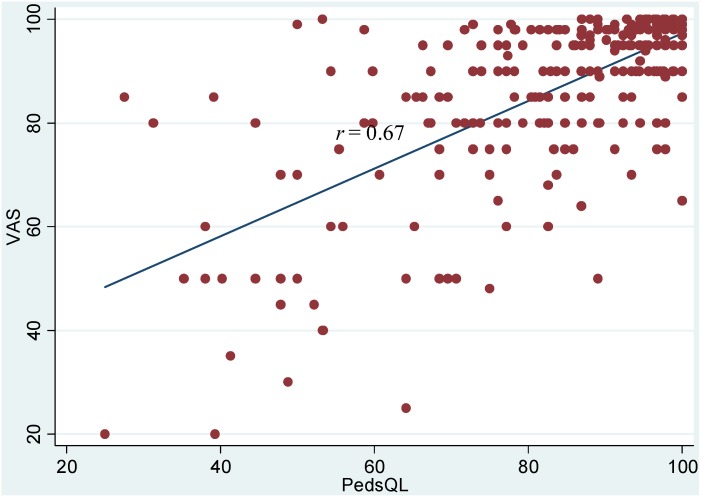
Scatter Plot and Fitted Line of VAS over PedsQL^TM^ Scores.

Table 1 includes descriptive statistics for the HRQoL measures and other study variables. The average score for VAS was 86.6 on scale from 0–100 (standard deviation of 15.9), while the average of PedsQL^TM^ was 83.8 (standard deviation of 16.4). Figure 1 shows a scatter plot of the VAS *vs*. PedsQL^TM^ scores along with their ordinary least squares (OLS) regression line. The two scores had a standardized correlation coefficient (*r*) of 0.67: a one standard deviation increase in PedsQL^TM^ was associated with a 0.67 standard deviation increase in VAS (and vice versa). This correlation is stronger than those previously reported between the PedsQL^TM^ and HRQoL instruments specific to oral health including the Child Oral Health Impact Profile when used among children and adolescents with oral clefts (*r* = 0.52) [20] and the Early Childhood Oral Health Impact Scale (*r* = 0.20) [21]. The stronger correlation suggests that the VAS is capturing more of the generic HRQoL measured by the PedsQL^TM^ compared to a condition-specific (*i.e.*, oral health) instrument. 

**Figure 2:**
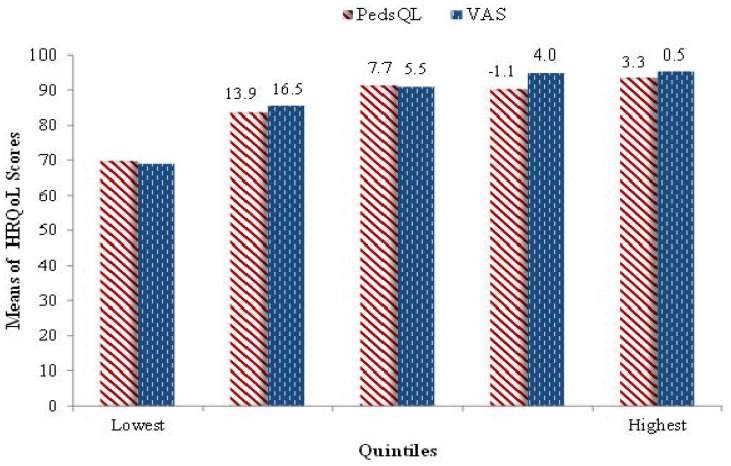
Means of VAS (PedsQL^TM^) Scores by Quintiles of PedsQL^TM^ (VAS).

Notes: The incremental changes in means of scores of one HRQL scale with moving to the next quintile of the other HRQL scale are shown at the top of the bars.

In Figure 2, we show the means of the VAS scores across quintiles of the PedsQL^TM^ and vice-versa. The VAS score means increased across the quintiles of the PedsQL^TM^ but the changes became smaller in magnitude with moving to successively higher quintiles. This was also generally the case for changes in the PedsQL^TM^ score means over the VAS quintile groups, with the exception that the PedsQL^TM^ score mean slightly declined between the third and fourth quintiles of the VAS. This indicates that the scores of the PedsQL^TM^ and VAS were overall more consistently related to each other at lower ranges, *i.e.*, for children with lower HRQoL, but were less so for children with high HRQoL.

Table 2 compares the correlations of measured indicators of child health and wellbeing that are thought to be relevant for HRQoL, one at a time, with each of VAS and PedsQL^TM^ scores. The correlations ranged from 0.20 to 0.54 (in absolute values) and were significant at *p* < 0.001. Overall, there were no prominent and consistent differences in the correlations of VAS and PedsQL^TM^ with these measures. The correlations were generally close with a difference between VAS and PedsQL^TM^ of less than 0.1 in all cases. No instrument clearly dominated the other one in being more strongly correlated with a greater number of the selected child health and wellbeing indicators.

**Table 2:** Correlations of VAS and PedsQL^TM^ with selected measures of child health and wellbeing.

Variable Name	Correlation with VAS	Correlation with PedsQL
Child’s overall health	−0.537	−0.538
chronic condition	−0.230 ^a^	−0.304
child frustrated because of trouble being understood	−0.355	−0.348
child avoids speaking	−0.388	−0.325
difficulty being understood by those who do see child often	−0.385	−0.324
difficulty being understood by those who do not see child much	−0.346	−0.342
happy with facial appearance	−0.256	−0.200 ^b^
social anxiety	−0.284	−0.225 ^a^
separation anxiety	−0.319	−0.366
PBS aggression/opposition	−0.366	−0.423
PBS hyperactivity/inattention	−0.379	−0.469
PBS depression/anxiety	−0.501	−0.482
PBS physical health	−0.420	−0.407

Notes: All correlations are significant at *p* < 0.00005 except where noted; ^a^ *p* = 0.0001; ^b^ *p* = 0.0006.

The HRQoL of children with oral clefts may vary by cleft type. However, the direction and magnitude of these differences are theoretically ambiguous. For example, it is unclear based on theory whether children with CLO have better or worse HRQoL than those with CPO. Even though speech problems are typically not present among children with CLO unlike those with cleft palate, both cleft types are associated with feeding problems and dental problems and cleft lip is additionally associated with esthetic concerns (and generally more dental issues). In order to evaluate how the two HRQoL scores compare by cleft type, we regressed using OLS the VAS and the PedsQL^TM^ scores on cleft type indicators including an indicator for CLO and another for CPO with CLP as the reference category (Table 3). Children with CLO had significantly higher PedsQL^TM^ scores than those with CLP; however, the difference in VAS scores was smaller and insignificant (*p* = 0.18). The difference between CPO and CLP was insignificant on both instruments. The difference between the instruments for CLO could suggest that the PedsQL^TM^ is more sensitive to identifying differences in HRQoL by cleft type. However, this difference could also be partly driven by the relatively small number of children in each cleft type and the skewed HRQoL score distributions (especially by cleft type) which could bias mean comparisons. When comparing the medians of the HRQoL scores between children with CLO and those with cleft palate (with or without cleft lip in one group, *i.e.*, combining CPO and CLP together), the VAS indicated higher HRQoL values among children with CLO; the difference in median scores between these two groups was slightly larger with the PedsQL^TM^ than VAS (7 *vs*. 5 points). Taken as a whole with the other results and considering the theoretical ambiguity about differences in HRQoL by cleft type and the relatively small number of children in each cleft type, differences between the instruments by cleft type do not necessarily suggest a weakness of the VAS in capturing overall HRQoL in this sample.

**Table 3:** Mean (OLS) and median regressions of VAS and PedsQL^TM^ scores on cleft type indicators.

Regressions	β (SE)	
	VAS	PedsQL^TM^
**Mean Regression (OLS)**		
CLO *vs.* CLP	3.08 (2.28)	6.14 (2.33) ***
CPO *vs.* CLP	−0.16 (2.16)	1.88 (2.21)
**Median Regression **		
CLO *vs.* CPO & CLP	5.00 (1.96) **	7.09 (2.54) ***

Notes: ** *p* < 0.05; ** *p* < 0.01.

## 4. Discussion

In this sample of children with oral clefts, the HRQoL scores from the VAS and PedsQL^TM^ were well-correlated and overall similar in their correlation with several health indicators. Given the simplicity of the VAS, it may be an appealing choice for cleft teams and other clinical providers of healthcare for children with clefts who may be primarily interested in screening children with oral clefts to identify those at risk for low HRQoL for more comprehensive evaluations instead of assessing specific HRQoL domains in every child. VAS may also be of interest to researchers of health services and outcomes among children with oral clefts who are mainly interested in measuring average HRQoL across a sample and those who are soliciting HRQoL values to generate QALYs for cost-effectiveness analysis. In contrast, one clear advantage of PedsQL^TM^ is in settings where clinicians or researchers are interested in decomposing total HRQoL across multiple domains to identify areas of functioning most adversely affected by the child’s health. 

There are other methods besides VAS to obtain HRQoL scores for QALY measurement in cost-effectiveness analysis such as the standard gamble (SG) and time trade-off (TTO). Both of these methods are rooted in economic theory, but they are not necessarily advantageous to VAS on either theoretical or empirical grounds [13]. Among the main theoretical limitations of these methods are their sensitivity and bias to preferences for risk taking (SG) and time/future discounting (TTO). On the practical side, these methods are particularly demanding on the raters’ cognitive ability and fairly burdensome (especially the SG) [8], typically requiring an interviewer and illustrative tools to aid the raters’ in their task. In contrast, the VAS can be easily self-administered after brief written instructions as done in this study. 

Our study has several strengths but some limitations. One strength is that mothers completed the PedsQL^TM^ and VAS at the same time so there is no timing bias due to changes in health status and no interviewer or data collection method bias since mothers self-administered both methods. Also, there was no language in the instructions that would alert the mothers to our objective of comparing the two methods and cause them to compare their own answers between these instruments. Furthermore, the Likert-scale answers to the PedsQL^TM^ questions are not directly comparable to the single VAS score and the total PedsQL^TM^ score derived from the answers is not available to the mothers. Therefore, it is unlikely that there is any bias in the correlation between the two scores due to the mode of administering the instruments. Another strength is that we measured several health indicators that we used to evaluate the sensitivity of the HRQoL scores and their ability to correlate with different aspects of health and wellbeing. 

On the limitation side, having other measures of the child’s health and well-being, such as measures of pain or cognitive performance, would have been useful to correlate within the quality of life measures. The correlations between the HRQoL scores and certain health/wellbeing indicators such as social anxiety and number of chronic conditions were relatively low. This is not surprising since the total scores are generic measures that capture overall HRQoL and how it is impacted by various physical and psychosocial aspects of health and wellbeing. The differences in correlations across the various health and wellbeing indicators may reflect the relative importance of these indicators for HRQoL. However, these results also highlight the value of domain-specific assessments and analyses which can be done with the PedsQL^TM^ in cases where specific areas of health and wellbeing such as physical or emotional functioning are of interest. We chose to measure overall HRQoL with the VAS in this study and therefore only compared the VAS to the overall PedsQL^TM^ scores instead of the domain- specific scores. However, future studies can evaluate the utility of VAS in specific domains of health and wellbeing. Also, we were unable to evaluate the test-retest and inter-rater reliability of the VAS for our study population and leave this for future research.

It is important to note that our results may not necessarily generalize to other conditions besides oral clefts such other birth defects or chronic health conditions. To the best of our knowledge, very few studies have reported the correlations between the PedsQL^TM^ and global measures of HRQoL using VAS in other pediatric populations, so there are not many previous results with which we can compare our finding. One study reported a correlation of 0.64 between the PedsQL^TM^ and a general VAS-based measure of wellbeing among children with chronic arthritis [22], which is close to the 0.67 correlation coefficient we found. Replicating this study in other pediatric populations may be of interest to further evaluate the value of VAS as a tool for HRQoL screening and assessment. 

## 5. Conclusions

Our study finds the VAS, a relatively simple technique, to perform relatively well in measuring overall HRQoL among children with oral clefts. The average VAS score of the sample was very close to the average PedsQL^TM^, the two scores were well-correlated (*r* = 0.67), and they were overall comparable in their correlation with several measures of child health and wellbeing. The VAS method may be particularly appealing to cleft teams and other health professionals providing care for children with oral clefts for screening children at risk of low HRQoL and referral into more comprehensive evaluations. This method may also be useful for researchers who are interested in measuring average HRQoL across a sample and generating HRQoL scores to obtain QALYs for cost-effectiveness analysis.
